# Aberrant HDAC3 expression correlates with brain metastasis in breast cancer patients

**DOI:** 10.1111/1759-7714.13561

**Published:** 2020-07-20

**Authors:** Li Ma, Lisha Qi, Shuangjing Li, Qiang Yin, Jinmei Liu, Jingyi Wang, Chunhua She, Peng Li, Qun Liu, Xiaoguang Wang, Wenliang Li

**Affiliations:** ^1^ Department of Neuro‐Oncology and Neurosurgery Tianjin Medical University Cancer Institute and Hospital, National Clinical Research Center for Cancer, Key Laboratory of Cancer Prevention and Therapy of Tianjin, Tianjin's Clinical Research Center for Cancer Tianjin China; ^2^ Department of Pathology Tianjin Medical University Cancer Institute and Hospital, National Clinical Research Center for Cancer, Key Laboratory of Cancer Prevention and Therapy of Tianjin, Tianjin's Clinical Research Center for Cancer Tianjin China; ^3^ Department of Central Laboratory Liaocheng People's Hospital Liaocheng China

**Keywords:** Brain metastasis, breast cancer, HDAC inhibitor, histone deacetylases

## Abstract

**Background:**

Brain metastasis is an unsolved clinical problem in breast cancer patients due to its poor prognosis and high fatality rate. Although accumulating evidence has shown that some pan‐histone deacetylase (HDAC) inhibitors can relieve breast cancer brain metastasis, the specific HDAC protein involved in this process is unclear. Thus, identifying a specific HDAC protein closely correlated with breast cancer brain metastasis will not only improve our understanding of the functions of the HDAC family but will also help develop a novel target for precision cancer therapy.

**Methods:**

Immunohistochemical staining of HDAC1, HDAC2, and HDAC3 in 161 samples from breast invasive ductal carcinoma patients, including 63 patients with brain metastasis, was performed using the standard streptavidin‐peroxidase method. The relationships between HDAC1, HDAC2, and HDAC3 and overall survival/brain metastasis‐free survival/post‐brain metastatic survival were evaluated using Kaplan‐Meier curves and Cox regression analyses.

**Results:**

HDAC1, HDAC2, and cytoplasmic HDAC3 all displayed typical oncogenic characteristics and were independent prognostic factors for the overall survival of breast cancer patients. Only cytoplasmic HDAC3 was an independent prognostic factor for brain metastasis‐free survival. Cytoplasmic expression of HDAC3 was further upregulated in the brain metastases compared with the matched primary tumors, while nuclear expression was downregulated. The HDAC1, HDAC2, and HDAC3 expression levels in the brain metastases were not correlated with survival post‐brain metastasis.

**Conclusions:**

Our studies first demonstrate a critical role for HDAC3 in the brain metastasis of breast cancer patients and it may serve as a promising therapeutic target for the vigorously developing field of precision medicine.

**Key points:**

Significant findings of the study

Cytoplasmic HDAC3 is an independent prognostic factor for the overall survival and brain metastasis‐free survival of breast cancer patients.

What this study adds

Cytoplasmic expression of HDAC3 was further upregulated in the brain metastases compared with the matched primary tumours, while nuclear expression was downregulated.

## Graphical abstract

Cytoplasmic HDAC3 is an independent prognostic factor for the overall survival and brain metastasis‐free survival of breast cancer patients.Cytoplasmic expression of HDAC3 was further upregulated in the brain metastases compared with the matched primary tumours, while nuclear expression was downregulated.

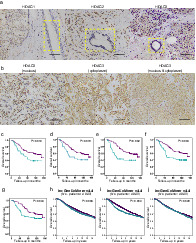

## Introduction

An estimated 20% of cancer patients will develop brain metastases, with the majority of brain metastases originating from lung cancer (20%–56% of patients), breast cancer (5%–20%), or melanoma (7%–16%).^1^, ^2^, ^3^ The incidence of brain metastasis (BM) in breast cancer patients is increasing year by year partly due to the rapid progress in the multimodal treatments of breast cancer; however, once brain metastasis occurs, the prognosis of these patients will remain very poor, with the two‐year survival rate in single digits.^4^, ^5^, ^6^ Although several genetic events, such as changes in ST6GALNAC5, CXCR4/CXCL12, and Slit2/Robo1, have been sporadically reported to correlate with breast cancer brain metastasis (BCBM) in the past few decades,^7^, ^8^, ^9^ the mechanisms underlying the central nervous system (CNS) relapse of breast cancer remain largely unknown.

Histone deacetylases (HDACs) play an important role in post‐translational modification in mammalian cells by removing acetyl groups from various histone and nonhistone proteins.^10^ To date, 18 histone deacetylases have been identified and categorized into four different classes (class I, II, III and IV) based on their homology.^11^ In tumorigenesis, the finely tuned acetylation status at the whole proteome level might be disrupted by dysregulated HDACs, and HDAC inhibitors could enable the re‐establishment of cellular acetylation‐deacetylation homeostasis, thus reversing cancer initiation and progression.^12^, ^13^ To date, numerous synthetic or natural inhibitors that target class I, II, and IV HDACs have been developed, most of which are pan inhibitors for two or more HDAC classes.^11^, ^14^ Accumulating evidence suggests that HDAC inhibitors can effectively relieve brain metastases from breast cancer. Palmieri *et al*. reported that the HDAC inhibitor vorinostat prevented the development of 231‐BR (a brain trophic subline of MDA‐MB‐231 human breast cancer cell line) micrometastases by 28% and large metastases by 62% compared with those in vehicle‐treated mice^15^; Kim *et al*. observed that the HDAC inhibitor SB939 reduced 4T1‐Br4 (a brain trophic subline of 4T1 mouse breast cancer cell line) metastasis to the brain in vivo and had potent radio‐sensitizing properties in vitro.^16^

Although increasing evidence has shown that pan‐HDAC inhibitors such as vorinostat and SB939 can reduce the occurrence of BCBM, it is still unclear which specific HDAC protein plays a key role in the process of breast cancer cell metastasis to the brain and whether such effect is directly caused by a particular HDAC protein or derived from HDAC downstream gene targets. In this study, we used a cohort of 161 patients with invasive ductal carcinoma (IDC) of the breast to investigate the specific roles of different HDAC proteins in BCBM. The related results will not only improve our understanding of the functions of the HDAC family but will also help develop more specific and efficient antitumour strategies based on a single therapeutic target. Since HDAC1, HDAC2, and HDAC3 are the most common histone deacetylases in human tissues with a relatively high abundance and have already been reported elsewhere to be potential oncogenes in breast cancer,^17^, ^18^ our studies mainly focus on these three HDAC proteins.

## Methods

### Breast Cancer patient selection and clinical information

A total of 63 IDC patients with brain metastasis, diagnosed between 2003 and 2018, were selected from the archives of the Pathology Department and Breast Pathology Department of Tianjin Medical University Cancer Institute and Hospital (TMUCIH). Paraffin‐embedded tissue chips of 98 patients with IDC but without brain metastasis were purchased from Shanghai Outdo Biotech Company (HBreD139Su01). A total of 139 primary breast tumors and 45 brain metastases were collected for HDAC immunohistochemical staining in our study. Among them, 24 were matched samples of primary and brain metastatic tumors from the same person. This study was reviewed and approved by the Ethics Committee of TMUCIH. The median age of the 161 patients with IDC was 51 years (range, 26–82), and they were all female. None had received neoadjuvant chemotherapy or preoperative radiation therapy. The patients were followed‐up for 2–158 months, and 79 (49.1%) patients died of tumors. Detailed information on the clinicopathological characteristics of the 161 patients can be found in Table S1.

### Immunohistochemical staining

Immunohistochemical staining of HDAC1, HDAC2, and HDAC3 was performed with the streptavidin‐peroxidase (S‐P) method as previously reported.^19^ Antigen retrieval was performed at 121°C for 2 minutes 30 seconds using citrate buffer (pH 6.0). After serial blocking with hydrogen peroxide and normal horse serum, the tissue chips and sections were incubated with primary monoclonal antibody against HDAC1 (cat. no. 10197‐1‐AP, Proteintech), HDAC2 (cat. no. 12922‐3‐AP, Proteintech) or HDAC3 (cat. no. 10255‐1‐AP, Proteintech) at 4°C overnight.

### Evaluation of staining

The tissue chips and sections stained immunohistochemically for HDAC1, HDAC2, and HDAC3 were reviewed and scored separately by two pathologists blinded to the clinical parameters. A third pathologist arbitrated any disagreements.

HDAC3 expression in the cytoplasm was evaluated according to the staining intensity and the percentage of positive cells, as we described previously.^20^ Staining intensity was measured and scored as follows: 0, negative; 1, weak; 2, moderate; 3, strong. The percentage of cells stained positively was scored as 0–100. Therefore, the total H score of cytoplasmic HDAC3 ranged from 0 to 300 by multiplying the intensity and the percentage scores. Because nuclear staining was present at a uniform intensity but to different extents, nuclear HDAC1, HDAC2, and HDAC3 expression was assessed by the percentage of positive nucleic‐stained cells and scored on a scale of 0 to 100. A cytoplasmic HDAC3 score of 20–300 was defined as C‐high and a score of 0–19 as C‐low; a nuclear HDAC3 score of 20–100 was defined as N‐high and a score of 0–19 as N‐low; 0–19 was defined as HDAC1/HDAC2 low, and 20–100 was defined as HDAC1/HDAC2 high.

### Statistical analysis

Overall survival (OS) was measured from the date of the breast cancer diagnosis to the date of death or the last follow‐up; brain metastasis‐free survival was defined as the time from the date of the breast cancer diagnosis to the date of the brain metastasis diagnosis; survival after brain metastasis was measured from the date of the brain metastasis diagnosis to the date of death or the last follow‐up. Survival outcomes were evaluated using the Kaplan‐Meier method, and differences between groups were compared by using log‐rank statistics. Quantitative data of HDAC1, HDAC2, and HDAC3 staining are expressed as the mean ± standard deviation (SD). Paired *t*‐tests were used to evaluate the statistical significance between matched samples of primary tumors and brain metastases, and unpaired *t*‐tests with Welch's correction were used to evaluate the differences of HDAC expression in patients with and without brain metastasis. The χ^2^ test was used to study the correlation between molecular subtypes and HDAC expression. Nonparametric Spearman's correlation analysis was used to assess the association between two variables. Univariate and multivariate Cox proportional hazards models were used to determine the associations of the clinicopathological parameters with survival outcomes. A variable with *P* < 0.10 in the univariate analysis was considered eligible for later multivariate analysis.

All reported *P*‐values were two‐sided, and differences reaching *P* < 0.05 were considered statistically significant. Statistical analyses were performed with IBM SPSS Statistics 19 and GraphPad Prism 7.

## Results

### HDAC1, HDAC2, and HDAC3 were upregulated in breast cancer tissues and correlated with worse prognosis in breast cancer patients

**Figure 1:**
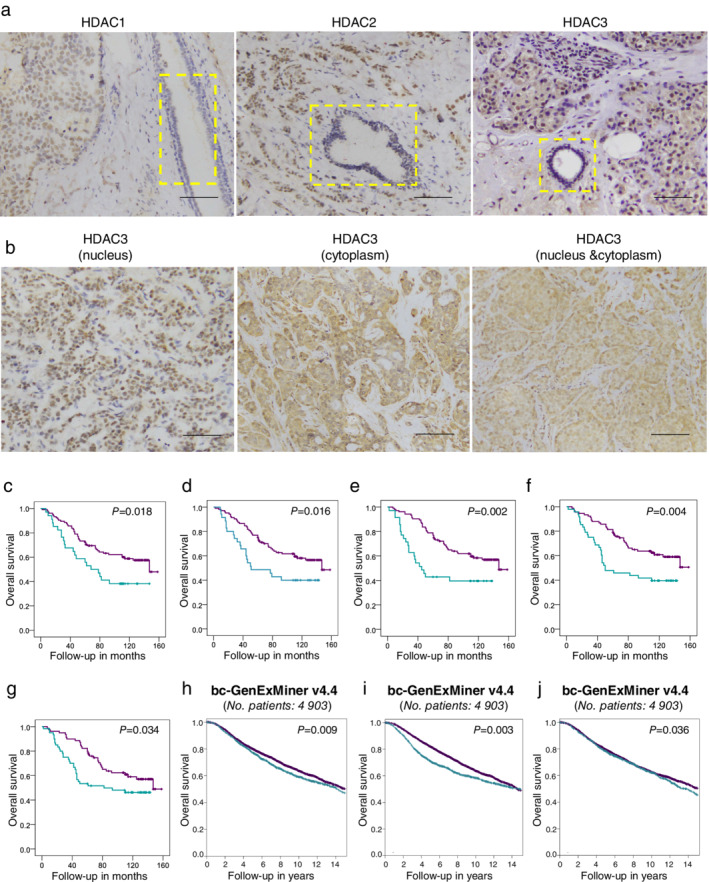
HDAC1, HDAC2, and HDAC3 were upregulated in breast cancer tissues and correlated with worse prognosis in breast cancer patients. (**a**) Representative immunohistochemical (IHC) staining photos of HDAC1, HDAC2, and HDAC3 in breast specimens. HDAC1, HDAC2, and HDAC3 levels were obviously elevated in the tumor tissues compared to the non‐neoplastic adjacent tissues of patients with invasive ductal carcinoma (IDC). Yellow boxes indicated mammary ducts. Scale bars, 100 μm. (**b**) Representative IHC photos of three different kinds of HDAC3 subcellular localization. Scale bars, 100 μm. (**c**–**f**) Overall survival (OS) curves of 139 IDC patients with different HDAC1 (**c**) (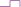) HDAC1 Low (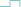) HDAC1 High (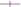) HDAC1 low‐censored (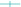) HDAC1 high‐censored, HDAC2 (**d**) (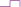) HDAC2 Low (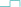) HDAC2 High (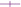) HDAC2 low‐censored (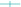) HDAC2 high‐censored, cytoplasmic HDAC3 (**e**) (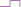) HDAC3 C‐low (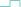) HDAC3 C‐high (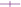) HDAC3 C‐low‐censored (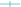) HDAC3 C‐high‐censored, and nuclear HDAC3 (**f**) levels (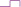) HDAC3 N‐low (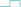) HDAC3 N‐high (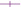) HDAC3 N‐low‐censored (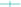) HDAC3 N‐high‐censored. According to another set of criteria in which cases with either high nuclear or cytoplasmic expression were classified into the C‐high/N‐high group and other cases were classified into the Others group, the overall survival curve of the 139 IDC patients was reproduced as Figure 1g (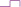) HDAC3 Others (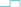) HDAC3 C‐high/N‐high (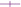) HDAC3 Others‐censored (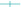) HDAC3 C‐high/N‐high‐censored. *P* values of the Kaplan‐Meier plots in (c‐g) were calculated by log‐rank test in IBM SPSS Statistics 19 software. (**h**–**j**) Kaplan‐Meier survival curves with log‐rank analysis were used to assess the correlation between HDAC1 (**h**) (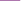) HDAC1 Low (≤ 75th percentile) (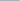) HDAC1 High (> 75th percentile), HDAC2 (**i**) (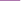) HDAC2 Low (≤ 75th percentile) (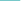) HDAC2 High (> 75th percentile), and HDAC3 (**j**) (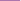) HDAC3 Low (≤ 80th percentile) (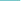) HDAC3 High (> 80th percentile) expression and overall survival of 4903 breast cancer patients in the bc‐GenExMiner platform (website: http://bcgenex.centregauducheau.fr; all DNA microarray data, node mixed, ER mixed; optimized split for HDAC1 and 2, an 80th percentile customized cutoff for HDAC3).

To identify whether histone deacetylases have direct relationships with the prognosis of breast cancer patients, we assessed the HDAC1, HDAC2, and HDAC3 protein expression data from a combined cohort of 161 breast cancer cases. Our results showed that the expression of HDAC1/HDAC2/HDAC3 increased significantly in the tumor tissues compared to the adjacent non‐neoplastic breast tissue (Fig 1a), indicating that the three histone deacetylases play vital roles in the initiation and development of breast cancer. Notably, HDAC1 and HDAC2 were predominantly located in the nucleus of breast cancer cells. However, the subcellular localization of HDAC3 varied in tumor cells and could be in the nucleus, cytoplasm, or both (Fig 1b). Therefore, the nuclear expression of HDAC1 and HDAC2 was scored and analyzed in the following study, while the nuclear and cytoplasmic expression of HDAC3 was studied separately in our study to ensure the accuracy and completeness of the results. The correlation between HDAC1, HDAC2, and HDAC3 expression and overall survival was investigated with Kaplan‐Meier survival analysis. As expected, the patients with higher HDAC1, HDAC2, nuclear HDAC3 and cytoplasmic HDAC3 usually had a worse prognosis (Fig 1c–f, all *P* < 0.05). To better match the evaluation criteria of HDAC3 in the bc‐GenExMiner database, which contained 4903 breast cancer patients (that database did not state whether HDAC3 was nuclear or cytoplasmic), we classified the cases with high nuclear or cytoplasmic expression of HDAC3 into the C‐high/N‐high group, and other cases were classified into the Others group. Accordingly, another survival curve of patients with different HDAC3 levels was produced as Fig 1g, and once again, patients with higher HDAC3 expression exhibited shorter overall survival (*P* < 0.05). We next investigated the relationship between HDAC expression and patient prognosis in the bc‐GenExMiner database. The trends in the above database were consistent with our previous conclusions; that is, higher HDAC1/HDAC2/HDAC3 expression often indicates a worse prognosis (Fig 1h–j, all *P* < 0.05). Thus, HDAC1, HDAC2, and HDAC3 all displayed typical oncogenic characteristics and were important in the early development of breast cancer and later survival of patients.

We also analyzed the roles of other common clinicopathological characteristics in the prognosis of those IDC patients in addition to HDAC1, HDAC2, and HDAC3. The univariate results of Cox regression analysis showed that increased tumor size/histological grade/lymph node invasion/HDAC1/HDAC2/nuclear HDAC3/cytoplasmic HDAC3 were significantly associated with shorter overall survival, while positive ER or PR was associated with prolonged overall survival (Table 1). In multivariate Cox regression analysis, HDAC1, HDAC2, or cytoplasmic HDAC3 was still an independent prognostic factor for the overall survival of breast cancer patients after correction for tumor size, histological grade, lymph node status, ER and PR (Table 1). Additionally, nuclear HDAC3 was not an independent prognostic factor in the multivariate analysis, suggesting that the influence of nuclear HDAC3 on the overall survival of such patients might be affected by some other variables.

**Table 1:** HDACs expression exhibited different roles in overall survival of IDC patients (*n* = 16l)

	Univariate		Multivariate		Multivariate		Multivariate		Multivariate	
Clinicopathological characteristics	HR(95%CI)	*P*‐value	HR(95%CI)	*P*‐value	HR(95%CI)	*P*‐value	HR (95%CI)	*P*‐value	HR (95%CI)	*P*
**Age, years**										
<48	1		/	/	/	/	/	/	/	/
≥48	1.398(0.892‐2.192)	0.144	/	/	/	/	/	/	/	/
**Tumor size** ^†^ **, cm**		0.000^*^		0.001^*^		0.004^*^		0.004^*^		0.002^*^
≤2	1		1		1		1		1	
2‐5	3.432(1.363‐8.640)	0.009^*^	4.918(1.165‐20.752)	0.030^*^	3.942(0.937‐16.581)	0.061	4.190(1.000‐17.565)	0.050	4.105(0.981‐17.188)	0.053
>5	10.957(4.141‐28.993)	0.000^*^	13.005(2.753‐61.445)	0.001^*^	9.130(1.966‐42.403)	0.005^*^	9.614(2.077‐44.510)	0.004^*^	9.998(2.177‐45.912)	0.003^*^
**Histological grade** ^†^		0.000^*^		0.000^*^		0.000^*^		0.000^*^		0.000^*^
I	1		1		1		1		1	
II	7.343(1.011‐53.346)	0.049^*^	3.966(0.518‐30.345)	0.184	3.283(0.433‐24.921)	0.250	3.578(0.472‐27.109)	0.217	3.076(0.403‐23.476)	0.279
III	32.775(4.422‐242.943)	0.001^*^	13.491(1.686‐107.931)	0.014^*^	10.961(1.377‐87.253)	0.024^*^	11.852(1.497‐93.838)	0.019^*^	10.884(1.363‐86.921)	0.024^*^
**Lymph node status** ^†^										
Negative	1		1		1		1		1	
Positive	1.952(1.182‐3.223)	0.009^*^	2.092(1.163‐3.764)	0.014^*^	1.781(0.985‐3.218)	0.056	1.697(0.936‐3.079)	0.082	1.572(0.862‐2.865)	0.140
**ER status** ^†^										
Negative	1		1		1		1		1	
Positive	0.303(0.190‐0.483)	0.000^*^	0.875(0.459‐1.669)	0.685	0.803(0.431‐1.498)	0.491	0.953(0.496‐1.833)	0.886	0.891(0.465‐1.705)	0.727
**PR status** ^†^										
Negative	1		1		1		1		1	
Positive	0.341(0.211‐0.550)	0.000^*^	0.515(0.269‐0.989)	0.046^*^	0.489(0.264‐0.906)	0.023^*^	0.455(0.239‐0.868)	0.017^*^	0.483(0.251‐0.930)	0.029^*^
**HER2 status** ^†^										
Negative	1		/	/	/	/	/	/	/	/
Positive	1.465(0.921‐2.332)	0.107	/	/	/	/	/	/	/	/
**HDAC1 status** ^†^										
Low	1		1							
High	2.540(1.549‐4.165)	0.000^*^	2.799(1.652‐4.741)	0.000^*^						
**HDAC2 status** ^†^										
Low	1				1					
High	1.874(1.111‐3.162)	0.019^*^			1.823(1.059‐3.136)	0.030^*^				
**HDAC3 status** ^†^ **(nucleus)**										
Low	1						1			
High	2.043(1.217‐3.430)	0.007^*^					1.645(0.941‐2.876)	0.081		
**HDAC3 status** ^†^ **(cytoplasm)**										
Low	1								1	
High	2.406(1.403‐4.126)	0.001^*^							1.948(1.089‐3.486)	0.025^*^

† Some data were missing.

* Indicates statistical significance (P < 0.05).

*P*‐value was calculated by Cox regression analysis.

### Patients with higher expression of HDAC3 exhibited earlier occurrence of brain metastasis (BM), and patients with BM had higher expression of HDAC3 than those without BM

The associations between HDAC1/HDAC2/HDAC3 and classical clinicopathological characteristics such as age, tumor size, histological grade, lymph node status, ER, PR, HER2, brain metastasis and molecular subtypes were analyzed by Spearman's rank‐correlation test and *χ* ^*2*^ test. As shown in Table 2, only cytoplasmic HDAC3 exhibited a significant correlation with histological grade, lymph node status, ER, molecular subtypes, and especially brain metastasis (*P* < 0.05).

**Table 2:** Relationship between clinicopathological characteristics and HDACs expression in IDC patients (*n* = 139)

		HDAC3 (cytoplasm), n				HDAC3 (nucleus), n				HDAC1, n				HDAC2, n			
Clinicopathological characteristics	Cases	Low	High	*r*_*s*_ or *χ* ^*2*^ ^‡^	*P* ^§^	Low	High	*r*_*s*_ or *χ* ^*2*^ ^‡^	*P* ^§^	Low	High	*r*_*s*_ or *χ* ^*2*^ ^‡^	*P* ^§^	Low	High	*r*_*s*_ or *χ* ^*2*^ ^‡^	*P* ^§^
**Age, years**				‐0.022	0.799			0.092	0.280			‐0.057	0.508			0.214	0.01^*^
<48	57	42	15			45	12			38	19			49	8		
≥48	82	62	20			58	24			59	23			55	27		
**Tumor size** ^†^ **, cm**				0.144	0.093			0.144	0.094			0.022	0.798			0.087	0.311
≤2	21	18	3			16	5			14	7			16	5		
2‐5	91	68	23			71	20			65	26			70	21		
>5	25	16	9			14	11			16	9			16	9		
**Histological grade** ^†^			0.203	0.018^*^		0.078	0.366			0.081	0.348v		0.079	0.362			
I	13	12	1			9	4			10	3			10	3		
II	97	74	23			75	22			68	29			74	23		
III	27	16	11			17	10			17	10			18	9		
**Lymph node status** ^†^				0.178	0.036^*^			0.155	0.069			‐0.006	0.948			0.006	0.940
Negative	52	44	8			43	9			36	16			39	13		
Positive	86	59	27			59	27			60	26			64	22		
**ER status** ^†^				‐0.186	0.029^*^			‐0.173	0.042^*^			‐0.066	0.445			0.030	0.724
Negative	44	28	16			28	16			29	15			34	10		
Positive	94	76	18			75	19			68	26			70	24		
**PR status** ^†^				‐0.092	0.283			‐0.043	0.619			‐0.114	0.183			‐0.024	0.775
Negative	62	44	18			15	17			40	22			46	16		
Positive	76	60	16			58	18			57	19			58	18		
**HER2 status** ^†^				0.077	0.371			0.064	0.457			‐0.042	0.625			0.112	0.191
Negative	90	70	20			69	21			62	28			71	19		
Positive	48	34	14			34	14			35	13			33	15		
**Brain metastasis**				0.279	0.001^*^			0.158	0.064			0.021	0.806			0.061	0.476
No	98	81	17			77	21			69	29			75	23		
Yes	41	23	18			26	15			28	13			29	12		
**Molecular subtypes** ^†^				7.670^¶^	0.022^*^ ^,^ ^¶^			4.453	0.108			1.859	0.395			0.699	0.705
Luminal	98	80	18			78	20			72	26			72	26		
HER2 overexpression	14	7	7			8	6			8	6			11	3		
Triple negative	26	17	9			17	9			17	9			21	5		

^†^ Some data were missing.

^‡^ *r*_*s*_ for age, tumor size, histological grade, lymph node status, ER, PR, HER2, and brain metastasis; *χ* ^*2*^ for molecular subtypes.

^§^ *P*‐value was calculated by Spearman's rank correlation test for age, tumor size, histological grade, lymph node status, ER, PR, HER2, and brain metastasis; *P*‐value was calculated by *χ* ^*2*^ test for molecular subtypes.

^¶^ HDAC3 (cytoplasm): Luminal vs. HER2 overexpression: *P* = 0.021, *χ* ^2^ = 5.363; Luminal versus Triple negative: *P* = 0.074, *χ* ^2^ = 3.185; HER2 overexpression versus Triple negative: *P* = 0.343, *χ* ^2^ = 0.897.

^*^ Indicates statistical significance (*P* < 0.05).

**Figure 2:**
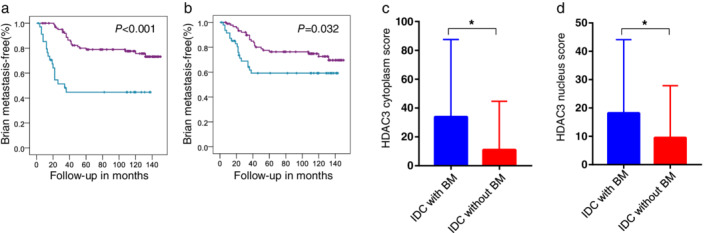
Patients with higher expression of HDAC3 exhibited earlier occurrence of brain metastasis (BM), and patients with BM have higher expression of HDAC3 than those without BM. (**a**–**b**) Brain metastasis‐free survival (BMF) curves of 139 IDC patients with different cytoplasmic HDAC3 (**a**) (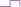) HDAC3 C‐low (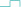) HDAC3 C‐high (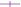) HDAC3 C‐low‐censored (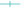) HDAC3 C‐high‐censored, and nuclear HDAC3 (**b**) levels (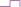) HDAC3 N‐low (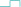) HDAC3 N‐high (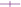) HDAC3 N‐low‐censored (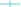) HDAC3 N‐high‐censored. *P*‐values of the Kaplan‐Meier plots in (**a**–**b**) were calculated by log‐rank tests in SPSS. (**c**–**d**) Cytoplasmic HDAC3 (**c**); and nuclear HDAC3 (**d**) IHC scores of IDC patients with BM (*n* = 41) and without BM (*n* = 98) were compared quantitatively and are shown as the mean ± SD visually. *P*‐values of (**c**–**d**) were calculated by unpaired *t*‐test with Welch's correction. ^*^ *P* < 0.05.

Kaplan‐Meier plots of brain metastasis‐free survival (BMF) in the same population further demonstrated that brain metastasis occurred earlier in those patients with high expression levels of HDAC3 (available for both subcellular locations, Fig 2a,b, all *P* < 0.05), while HDAC1 and HDAC2 again were not associated with brain metastases (Fig S1a and S1b).

Unsurprisingly, cytoplasmic HDAC3 in the patients with brain metastasis was much higher than that in the patients without brain metastasis (Fig 2c, with vs. without: 33.900 ± 8.373 vs. 11.020 ± 3.403, *P* = 0.014); nuclear HDAC3 was also higher in the patients with brain metastasis than in those without brain metastasis (Fig 2d, with vs. without: 18.170 ± 4.049 vs. 9.235 ± 1.771, *P* = 0.048); there was no significant difference in the average score or distribution of HDAC1/HDAC2 between these two groups of patients (Fig S1c and S1d).

The univariate results of Cox regression analysis showed that increased tumor size/nuclear HDAC3/cytoplasmic HDAC3 were significantly associated with earlier occurrence of brain metastasis, while positive ER or PR was associated with delayed brain metastasis (Table 3). In multivariate Cox regression analysis, cytoplasmic HDAC3 was still an independent prognostic factor for the occurrence of brain metastasis in the breast cancer patients after correction for tumor size, ER, PR, and HER2 (Table 3); nuclear HDAC3 was not an independent prognostic factor in the multivariate analysis, which suggested that the influence of nuclear HDAC3 on brain metastasis in such patients was also possibly linked with other variables.

**Table 3:** HDACs expression exhibited different roles in the onset of brain metastasis of IDC patients (*n* = 161)

	Univariate		Multivariate		Multivariate	
Clinicopathological characteristics	HR (95% CI)	*P*‐value	HR (95% CI)	*P*‐value	HR (95% CI)	*P*‐value
**Age, years**						
<48	1		/	/	/	/
≥48	0.760(0.463‐1.250)	0.280	/	/	/	/
**Tumor size** ^**†**^ **, cm**		0.000^*^		0.006^*^		0.001^*^
≤2	1		1		1	
2‐5	1.409(0.647‐3.070)	0.388	2.576(0.599‐11.086)	0.204	2.550(0.597‐10.900)	0.207
>5	5.159(2.220‐11.986)	0.000^*^	7.317(1.569‐34.120)	0.011^*^	8.975(1.953‐41.239)	0.005^*^
**Lymph node status** ^*^						
Negative	1		/	/	/	/
Positive	1.466(0.858‐2.504)	0.161	/	/	/	/
**ER status** ^**†**^						
Negative	1		1		1	
Positive	0.279(0.165‐0.471)	0.000^*^	0.499(0.216‐1.153)	0.104	0.541(0.231‐1.266)	0.157
**PR status** ^**†**^						
Negative	1		1		1	
Positive	0.360(0.211‐0.614)	0.000^*^	0.448(0.190‐1.054)	0.066	0.417(0.164‐1.058)	0.066
**HER2 status** ^**†**^						
Negative	1		1		1	
Positive	1.655(0.984‐2.783)	0.057	1.264(0.642‐2.487)	0.497	1.045(0.521‐2.093)	0.902
**HDAC1 status** ^**†**^						
Low	1					
High	1.370(0.705‐2.661)	0.353				
**HDAC2 status** ^**†**^						
Low	1					
High	1.555(0.793‐3.051)	0.199				
**HDAC3 status** ^**†**^ **(nucleus)**						
Low	1		1			
High	2.305(1.219‐4.360)	0.010^*^	1.712(0.843‐3.477)	0.137		
**HDAC3 status** ^**†**^ **(cytoplasm)**						
Low	1				1	
High	3.932(2.112‐7.322)	0.000^*^			3.386(1.724‐6.650)	0.000^*^

^†^ Some data were missing.

^*^ Indicates statistical significance (*P* < 0.05).

*P*‐value was calculated by Cox regression analysis.

### Cytoplasmic expression of HDAC3 upregulated in brain metastasis specimens compared with matched primary tumor specimens and nuclear HDAC3 expression was inversely downregulated

**Figure 3:**
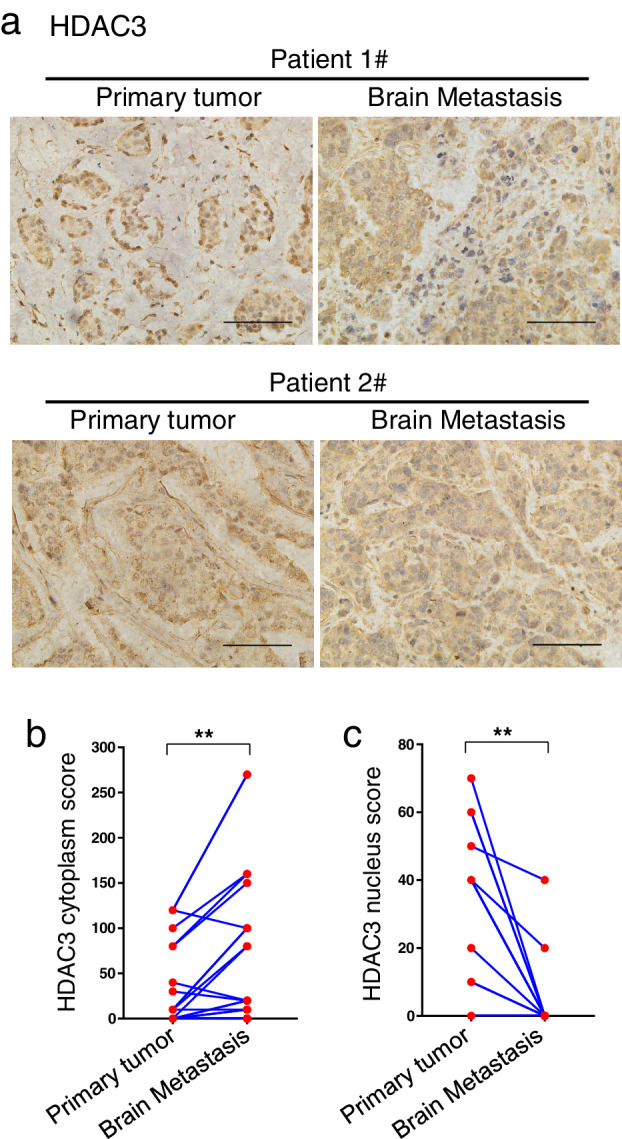
Cytoplasmic expression of HDAC3 was further upregulated in brain metastasis specimens compared with the matched primary tumor specimens, while nuclear HDAC3 expression was inversely downregulated. (**a**) Representative immunohistochemical (IHC) staining photos of HDAC3 in primary breast tumor sites and matched brain metastasis specimens were shown. Photos were taken at a magnification of 400×. Scale bars, 100 μm. (**b**–**c**) Cytoplasmic HDAC3 (**b**) and nuclear HDAC3 (**c**) IHC scores of 24 primary breast tumors and matched brain metastases are shown by symbols and lines plot, and *P*‐values were calculated by paired *t*‐tests (scores overlapped in some cases). ^**^ *P* < 0.01.

Representative immunohistochemical staining images of HDAC3 in the primary breast tumors and their matched brain metastases are shown in Fig 3a. The results visually demonstrated that the concentrated area of positive HDAC3 staining changed from the nucleus in the primary tumors to the cytoplasm in the matched brain metastases in these two cases. The changes in cytoplasmic and nuclear HDAC3 scores of the matched samples are shown in Fig 3b,c on a case‐by‐case basis (note: scores and lines overlapped in some cases). The cytoplasmic HDAC3 scores in the brain metastases were significantly higher than those in the matched primary tumors (Fig 3b, *P* = 0.002), while the nuclear HDAC3 scores changed in the opposite direction (Fig 3c, *P* = 0.003). There were two possible explanations for the above results: (i) HDAC3 protein transferred from the nucleus to the cytoplasm during breast cancer cell establishment in the brain, which indicated that the biological function of HDAC3 in cells of this subtype might have changed; (ii) a subset of breast cancer cells with high cytoplasmic expression of HDAC3 in primary tumors had a higher metastatic potential, and therefore, such cells were enriched in the brain metastases.

There were no significant differences in HDAC1 and HDAC2 expression between the primary tumors and their matched brain metastases (Fig S2a,b).

### HDAC1, HDAC2, and HDAC3 expression levels in brain metastases were not correlated with survival after brain metastasis in IDC patients

**Figure 4:**
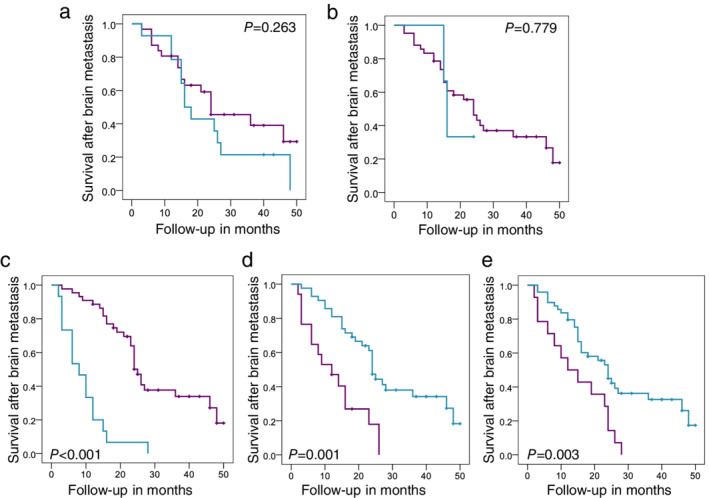
Survival after brain metastasis of IDC patients was not correlated with HDAC3 expression in brain metastases; however, no meningeal metastasis complication, CNS surgery, or CNS radiotherapy was associated with a better patient prognosis. (**a**–**e**) The relationship between cytoplasmic HDAC3 (**a**, expression of brain metastases, *n* = 45) (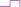) HDAC3 C‐low (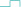) HDAC3 C‐high (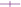) HDAC3 C‐low‐censored (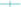) HDAC3 C‐high‐censored, nuclear HDAC3 (**b**, expression of brain metastases, *n* = 45) (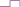) HDAC3 N‐low (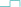) HDAC3 N‐high (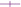) HDAC3 N‐low‐censored (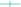) HDAC3 N‐high‐censored, meningeal metastasis complication (**c**, *n* = 59) (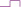) without meningeal metastasis (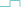) with meningeal metastasis (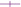) without meningeal metastasis‐censored (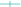) with meningeal metastasis‐censored, CNS surgery (**d**, *n* = 63), or CNS radiotherapy (e, *n* = 59) (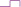) without CNS radiotherapy (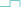) with CNS radiotherapy (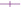) without CNS radiotherapy‐censored (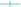) with CNS radiotherapy‐censored and survival after brain metastasis of IDC patients were shown in the corresponding Kaplan‐Meier plots CNS surgery (d, *n*=63). *P*‐values were calculated by log‐rank tests in SPSS.

We next explored whether HDACs in brain metastases were correlated with prognosis after brain metastasis. Kaplan‐Meier survival analysis showed that the HDAC1, HDAC2, and HDAC3 expression levels all had no significant effect on the post‐brain metastatic survival of 45 IDC patients (Fig S3a,b, Fig 4a,b, all *P* > 0.05).

The univariate results of Cox regression analysis showed that receiving CNS surgery or radiotherapy could prolong the post‐brain metastatic survival of these patients, while the occurrence of meningeal metastasis complication shortened their post‐brain metastatic survival (Table 4); HDAC1, HDAC2, and HDAC3 expression levels and other clinical characteristics, such as age, number of brain metastases and extracranial metastases, were not correlated with the prognosis after brain metastasis, and they were not included in the multivariate analysis (Table 4, all *P* > 0.1). In multivariate Cox regression analysis, CNS radiotherapy and meningeal metastasis complication were still independent prognostic factors for the post‐brain metastatic survival of these IDC patients (Table 4). The Kaplan‐Meier plots more intuitively demonstrated the effects of meningeal metastasis complication, CNS surgery and radiotherapy on the post‐brain metastatic survival (Fig 4c–e, all *P* < 0.01, median survival time: with meningeal metastasis versus without meningeal metastasis = 8 months versus 25 months; with CNS surgery versus without CNS surgery = 24 months versus 12 months; with CNS radiotherapy versus without CNS radiotherapy = 24 months versus 12 months).

**Table 4:** The roles of HDACs expression and other clinicopathological characteristics played in the prognosis of breast cancer patients after brain metastasis (*n* = 63)

	Univariate		Multivariate	
Clinicopathological characteristics	HR (95%CI)	*P*‐value	HR (95%CI)	*P*‐value
**Age, years**				
<48	1			
≥48	1.278(0.708‐2.305)	0.416		
**Number of brain metastases** ^†^				
Single	1			
Multiple	1.423(0.726‐2.792)	0.305		
**Extracranial metastases (lung, liver, bone)** ^†^				
No	1			
Yes	1.211(0.654‐2.239)	0.543		
**Meningeal metastasis complication** ^†^				
No	1		1	
Yes	5.944(2.984‐11.843)	0.000^*^	4.568(2.107‐9.907)	0.000^*^
**CNS surgery**				
No	1		1	
Yes	0.405(0.212‐0.772)	0.006^*^	0.714(0.343‐1.484)	0.367
**CNS radiotherapy** ^†^				
No	1		1	
Yes	0.293(0.150‐0.575)	0.000^*^	0.346(0.174‐0.690)	0.003^*^
**HDAC1 status of brain metastases** ^†^				
Low	1			
High	1.496(0.689‐3.250)	0.309		
**HDAC2 status of brain metastases** ^†^				
Low	1			
High	1.125(0492‐2.571)	0.780		
**HDAC3 status (nucleus) of brain metastases** ^†^				
Low	1			
High	1.224(0.286‐5.236)	0.785		
**HDAC3 status (cytoplasm) of brain metastases** ^†^				
Low	1			
High	1.509(0.719‐3.166)	0.277		

^†^ Some data were missing.

^*^ Indicates statistical significance (*P* < 0.05).

*P*‐value was calculated by Cox analysis.

## Discussion

To date, the mechanisms responsible for the brain colonization of breast cancer cells remain poorly understood and little investigated, partly due to its relatively low incidence and the complexity of the blood‐brain barrier (BBB).^4^, ^21^ Inspired by the positive results of pan‐HDAC inhibitors in the research field of BCBM, we used a cohort of 161 patients with IDC to investigate the specific roles of different HDAC proteins in the process of BCBM. We found that although HDAC1, HDAC2, and HDAC3 all displayed typical oncogenic characteristics in the tumorigenesis and development of breast cancer, only patients with high expression of HDAC3 exhibited earlier occurrence of brain metastasis (BM), and the patients with BM had relatively higher HDAC3 expression than those without BM. Moreover, cytoplasmic expression of HDAC3 was upregulated, while nuclear HDAC3 expression was inversely downregulated in brain metastatic tumors. These results indicate that HDAC3 probably participates in not only the early phase, but also the middle and late stages of BCBM.

HDAC3 possesses a unique property among the class I family of HDACs, as it is able to shuttle between the nucleus and the cytoplasm, whereas the other family members (HDAC1, HDAC2, and HDAC8) are found primarily in the nucleus.^22^, ^23^ HDAC3 contains one nuclear localization signal (NLS) and two different nuclear export sequences (NES).^23^, ^24^ The biological function of HDAC3 varies with protein localization and cell type. For example, Park *et al*. reported that the nuclear localization of HDAC3 was essential for the expression regulation of MDR1 in drug‐resistant cancer cell lines,^25^ and Escaffit *et al*. demonstrated a crucial role of cytoplasmic HDAC3 in the apoptosis progression in Jurkat and U2OS cells.^23^ Herein, our studies first reveal that HDAC3 is closely related to the occurrence and development of brain metastasis in breast cancer patients, and moreover, the cytoplasmic expression of HDAC3 may play a more important role than nuclear expression in the metastatic process.

However, in our study, the HDAC3 expression level in the brain metastases was not correlated with post‐brain metastatic survival. One plausible explanation is that survival after brain metastasis is influenced by many factors,^26^ such as the anatomical location of the brain metastasis, control of extracranial metastases, and the myriad of treatment options available; thus, the role of HDAC3 on post‐brain metastatic survival is very likely to be overridden. Another possible reason is that the sample size of our study is still relatively small; if more breast cancer patients with brain metastasis are included, the effect of cytoplasmic HDAC3 on post‐brain metastatic survival may become statistically significant.

Finally, we did not discuss the relationship between HDACs and extracranial metastasis of breast cancer in this article due to lack of complete metastasis information of patients in the tissue chips. Breast cancer has a greater chance of metastasizing to the lung and bone than to the brain,^4^, ^21^ and among the IDC patients with brain metastasis in our study, 49.2% (29/59) had at least one extracranial metastasis (unpublished data). Zhou *et al*. reported that HDAC inhibitors such as psammaplins and trichostatin A could disrupt the organ‐tropic (lung, bone, and brain) metastasis of MDA‐MB‐231 sublines.^27^ Thus, whether HDAC3 is specifically associated with brain metastasis, or is associated with multiple organ metastases in breast cancer patients requires further study.

## Disclosure

The authors declare that there are no potential conflicts of interest.

## Supporting information


**Table S1** The clinicopathological characteristics of 161 IDC patients.

**Figure S1** HDAC1 & HDAC2 exhibited no statistically significant correlation with brain metastasis of IDC patients. (**a**–**b**) Brain metastasis‐free survival (BMF) curves of 139 IDC patients with different HDAC1 (**a**) and HDAC2 (**b**) levels. *P*‐value of the Kaplan‐Meier plots in (**a**–**b**) were calculated by log‐rank test in SPSS. (**c**–**d**) HDAC1 (**c**) and HDAC2 (**d**) IHC scores of IDC patients with BM (*n* = 41) and without BM (*n* = 98) were compared quantitatively and shown by mean ± SD visually. *P*‐value of (**c**–**d**) were calculated by unpaired *t*‐test with Welch's correction, both *P* > 0.05.
**Figure S2** There were no significant differences of HDAC1 & HDAC2 expression between primary tumors and brain metastases of IDC patients. (**a**–**b**) HDAC1 (**a**) and HDAC2 (**b**) IHC scores of 24 primary breast tumors and matched brain metastases were shown by symbols and lines plot, and *P*‐values were calculated by paired *t*‐test (scores were overlapped in some cases, both *P* > 0.05).
**Figure S3** HDAC1 or HDAC2 expression in brain metastases was not correlated with the survival after brain metastasis of IDC patients. (**a**–**b**) The relationship between HDAC1 (**a**, expression of brain metastases, *n* = 45), HDAC2 (**b**, expression of brain metastases, *n* = 45) and survival after brain metastasis of IDC patients were shown in corresponding Kaplan‐Meier plots, and *P*‐value were calculated by log‐rank test in SPSS.
