# Supplementary figures and images for: Aberrant HDAC3 expression correlates with brain metastasis in breast cancer patients
Source: Thorac Cancer. 2020 Jul 20;11(9):2493–505. doi: 10.1111/1759-7714.13561 (PMC7471029; doi:10.1111/1759-7714.13561)

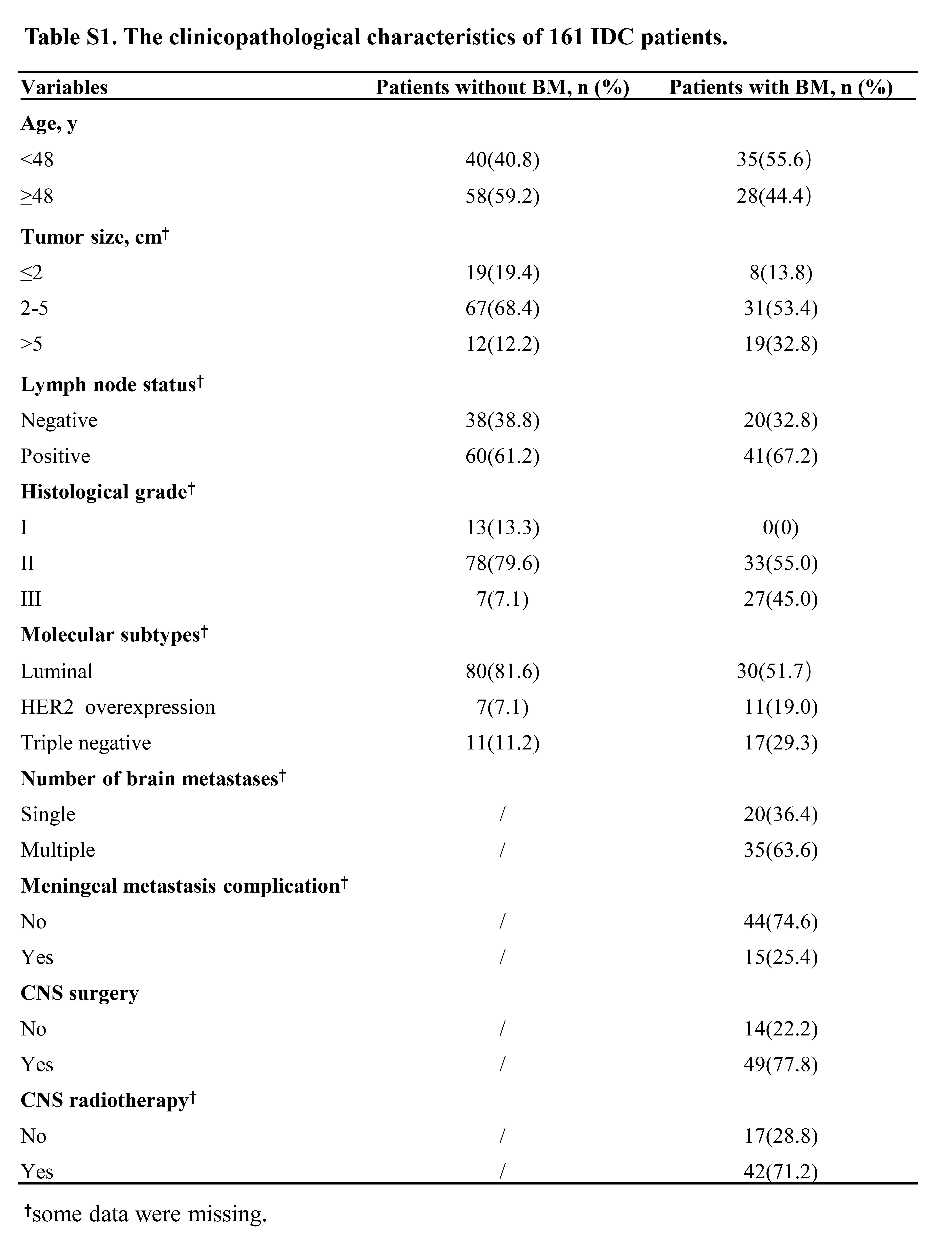

Supplement: Supplementary file 1 — Table S1 The clinicopathological characteristics of 161 IDC patients. [file TCA-11-2493-s001.tif]
