# Supplementary material for: Aberrant HDAC3 expression correlates with brain metastasis in breast cancer patients
Source: Thorac Cancer. 2020 Jul 20;11(9):2493–505. doi: 10.1111/1759-7714.13561 (PMC7471029; doi:10.1111/1759-7714.13561)
Supplement: Supplementary file 2 — Figure S1 HDAC1 & HDAC2 exhibited no statistically significant correlation with brain metastasis of IDC patients. (a–b) Brain metastasis‐free survival (BMF) curves of 139 IDC patients with different HDAC1 (a) and HDAC2 (b) levels. P‐value of the Kaplan‐Meier plots in (a–b) were calculated by log‐rank test in SPSS. (c–d) HDAC1 (c) and HDAC2 (d) IHC scores of IDC patients with BM (n = 41) and without BM (n = 98) were compared quantitatively and shown by mean ± SD visually. P‐value of (c–d) were calculated by unpaired t‐test with Welch's correction, both P > 0.05. Figure S2 There were no significant differences of HDAC1 & HDAC2 expression between primary tumors and brain metastases of IDC patients. (a–b) HDAC1 (a) and HDAC2 (b) IHC scores of 24 primary breast tumors and matched brain metastases were shown by symbols and lines plot, and P‐values were calculated by paired t‐test (scores were overlapped in some cases, both P > 0.05). Figure S3 HDAC1 or HDAC2 expression in brain metastases was not correlated with the survival after brain metastasis of IDC patients. (a–b) The relationship between HDAC1 (a, expression of brain metastases, n = 45), HDAC2 (b, expression of brain metastases, n = 45) and survival after brain metastasis of IDC patients were shown in corresponding Kaplan‐Meier plots, and P‐value were calculated by log‐rank test in SPSS. [file TCA-11-2493-s002.pdf]

Table S1. The clinicopathological characteristics of 161 IDC patients.

Variables	Patients without BM, n (%)	Patients with BM, n (%)
**Age, y**		
<48	40(40.8)	35(55.6)
≥48	58(59.2)	28(44.4)
**Tumor size, cm†**		
≤2	19(19.4)	8(13.8)
2-5	67(68.4)	31(53.4)
>5	12(12.2)	19(32.8)
**Lymph node status†**		
Negative	38(38.8)	20(32.8)
Positive	60(61.2)	41(67.2)
**Histological grade†**		
I	13(13.3)	0(0)
II	78(79.6)	33(55.0)
III	7(7.1)	27(45.0)
**Molecular subtypes†**		
Luminal	80(81.6)	30(51.7)
HER2 overexpression	7(7.1)	11(19.0)
Triple negative	11(11.2)	17(29.3)
**Number of brain metastases†**		
Single	/	20(36.4)
Multiple	/	35(63.6)
**Meningeal metastasis complication†**		
No	/	44(74.6)
Yes	/	15(25.4)
**CNS surgery**		
No	/	14(22.2)
Yes	/	49(77.8)
**CNS radiotherapy†**		
No	/	17(28.8)
Yes	/	42(71.2)

†some data were missing.

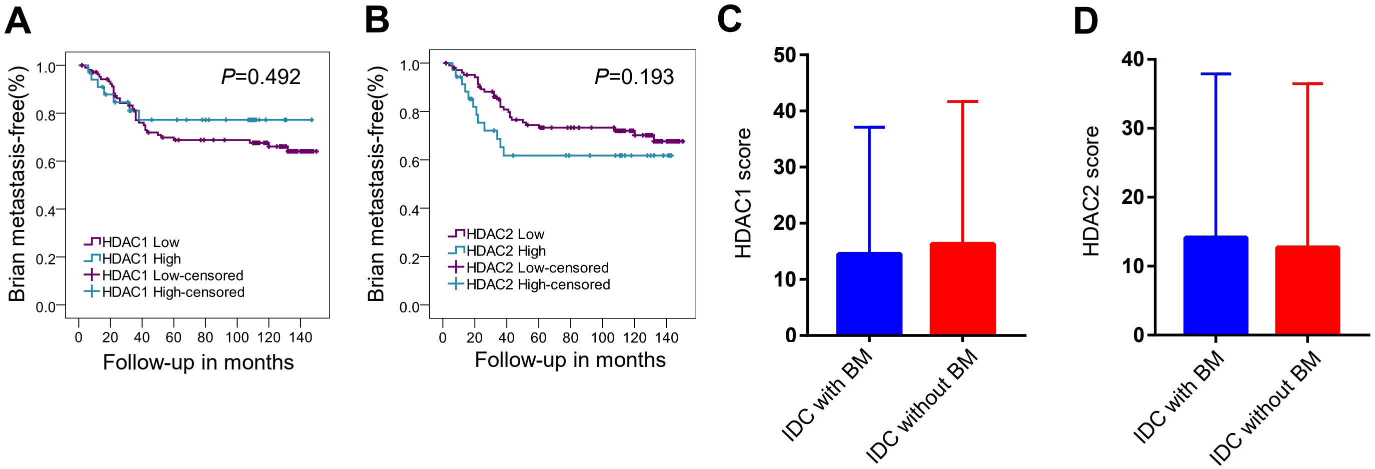

Figure S1. HDAC1 & HDAC2 exhibited no statistically significant correlation with brain metastasis of IDC patients. (A)-(B): Brain metastasis-free survival (BMF) curves of 139 IDC patients with different HDAC1(A) and HDAC2(B) levels. *P* value of the Kaplan-Meier plots in (A)-(B) were calculated by log-rank test in SPSS. **(C)-(D)**: HDAC1 (C) and HDAC2 (D) IHC scores of IDC patients with BM (n=41) and without BM(n=98) were compared quantitatively and shown by mean ± SD visually. *P* value of (C)-(D) were calculated by unpaired t test with Welch‘s correction, both $P > 0.05$.

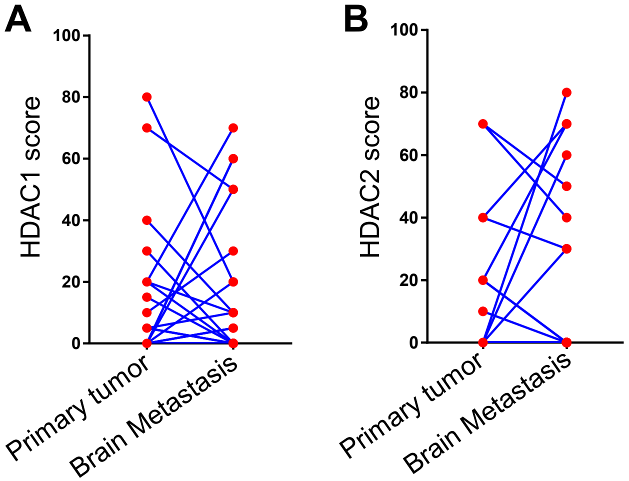

Figure S2. There were no significant differences of HDAC1 & HDAC2 expression between primary tumors and brain metastases of IDC patients.

(A)-(B): HDAC1(A) and HDAC2(B) IHC scores of 24 primary breast tumors and matched brain metastases were shown by symbols & lines plot, and *P* values were calculated by paired t test (scores were overlapped in some cases, both $P > 0.05$).

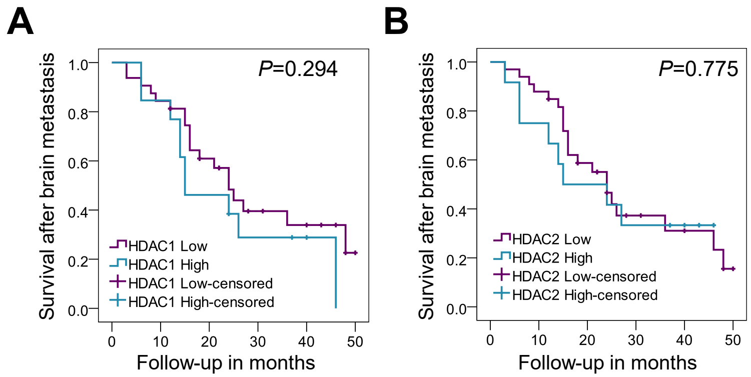

Figure S3. HDAC1 or HDAC2 expression in brain metastases was not correlated with the survival after brain metastasis of IDC patients. **(A)-(B)**:The relationship between HDAC1(A, expression of brain metastases, n=45), HDAC2(B, expression of brain metastases, n=45) and survival after brain metastasis of IDC patients were shown in corresponding Kaplan-Meier plots, and *P* value were calculated by log-rank test in SPSS.
